# Community Transmission of SARS-CoV-2 Associated with a Local Bar Opening Event — Illinois, February 2021

**DOI:** 10.15585/mmwr.mm7014e3

**Published:** 2021-04-09

**Authors:** Samira Sami, Caitlin R. Turbyfill, Shelby Daniel-Wayman, Stacy Shonkwiler, Kiva A. Fisher, Macey Kuhring, Aaron M. Patrick, Stephanie Hinton, Amanda S. Minor, Jessica N. Ricaldi, Ngozi Ezike, Judy Kauerauf, Wayne A. Duffus

**Affiliations:** ^1^CDC COVID-19 Response Team; ^2^Illinois Department of Public Health; ^3^Douglas County Department of Public Health, Tuscola, Illinois.

During February 2021, an opening event was held indoors at a rural Illinois bar that accommodates approximately 100 persons. The Illinois Department of Public Health (IDPH) and local health department staff members investigated a COVID-19 outbreak associated with this opening event. Overall, 46 COVID-19 cases were linked to the event, including cases in 26 patrons and three staff members who attended the opening event and 17 secondary cases. Four persons with cases had COVID-19–like symptoms on the same day they attended the event. Secondary cases included 12 cases in eight households with children, two on a school sports team, and three in a long-term care facility (LTCF). Transmission associated with the opening event resulted in one school closure affecting 650 children (9,100 lost person-days of school) and hospitalization of one LTCF resident with COVID-19. These findings demonstrate that opening up settings such as bars, where mask wearing and physical distancing are challenging, can increase the risk for community transmission of SARS-CoV-2, the virus that causes COVID-19. As community businesses begin to reopen, a multicomponent approach should be emphasized in settings such as bars to prevent transmission* (*1*). This includes enforcing consistent and correct mask use, maintaining ≥6 ft of physical distance between persons, reducing indoor bar occupancy, prioritizing outdoor seating, improving building ventilation, and promoting behaviors such as staying at home when ill, as well as implementing contact tracing in combination with isolation and quarantine when COVID-19 cases are diagnosed.

## Investigation and Findings

On February 17, 2021, IDPH was notified through the state’s outbreak reporting system of a possible COVID-19 outbreak (i.e., five or more cases linked to a common location) in persons who attended an opening event at a bar in a rural Illinois county. The event had occurred indoors, with no outside air flow, approximately 2 weeks earlier in a 2,800-sq-ft bar during normal operating hours (4:00 p.m. to 1:00 a.m.). Six employees staffed the bar. Although the total number of bar patrons who attended the event is unknown, the bar accommodates approximately 100 persons. Before the event, IDPH reported a 7-day average daily COVID-19 incidence of 41–42 cases per 100,000 persons in the county; 14 days after the event, the 7-day average daily incidence had more than doubled, to 86–87 cases per 100,000 persons (*2*). On February 12, through routine testing and contact tracing, local health department staff members identified a cluster of cases linked to the bar event, including a case in an asymptomatic attendee who received a confirmed COVID-19 diagnosis the day before the event.

A bar attendee case was defined as the onset of COVID-19–like symptoms or receipt of a positive SARS-CoV-2 test result within 14 days of the bar opening event in a bar patron or employee who reported attending the event and who had no previous identified epidemiologic link to a COVID-19 case outside that setting. A confirmed case was defined as receipt of a positive SARS-CoV-2 nucleic acid amplification test (NAAT) or antigen test result by a person who attended the event, and a probable case was defined as COVID-19–like symptoms in a person who attended the event but had no laboratory confirmation of infection.^†^ A secondary case was defined as receipt of a positive SARS-CoV-2 NAAT or antigen test result by a close contact of a person with event-associated COVID-19.^§^

Local health department staff members, per standard practice, conducted case investigations within 48 hours of receipt of a positive SARS-CoV-2 test result in the county using a standardized questionnaire; demographic data, symptoms, and symptom onset date were entered into an electronic contact tracing platform. Through routine case investigation, local health department investigators identified a cluster of cases linked to the bar opening event through case reports indicating that persons attended the event or were close contacts of a person with an event-associated case during the 14 days before symptom onset or the testing date. All persons with a bar attendee case or secondary case of COVID-19 were interviewed by local health department staff members. This activity was reviewed by CDC and was conducted consistent with applicable federal law and CDC policy.^¶^

**Bar patrons and employees.** By February 16, 2021, 29 bar attendee cases had been identified among persons who reported attending the opening event (Figure), including 26 (89.7%) in bar patrons and three (10.3%) in employees; all identified cases were confirmed by NAAT or antigen testing, except one probable case in a person who had COVID-19–like symptoms but did not receive testing (Table). Three additional employees worked during the event, all of whom had received a positive test result during the preceding 90 days and had completed 10 days of isolation from symptom onset or test date. Among persons with bar attendee cases, 25 (86.2%) had symptomatic illnesses. Among persons with symptom onset after the start of the event, onset dates ranged from 1 to 7 days after the event. Four (13.8%) persons with bar attendee cases reported having symptoms on the day of the event and were not reported to be contacts of one another before the event. Event attendees reported inconsistent mask use and not maintaining ≥6 ft of physical distance, despite table spacing and signs encouraging physical distancing and mask use. Most persons with bar attendee cases were adults aged 18–44 years (75.9%), male (65.5%), and non-Hispanic White persons (79.3%). One of the 29 persons with a bar attendee case, a bar patron, had received a COVID-19 vaccination before the event (the first dose, 5 days before receipt of the positive SARS-CoV-2 test result). No other persons with bar attendee cases had received a COVID-19 vaccination.

**FIGURE:**
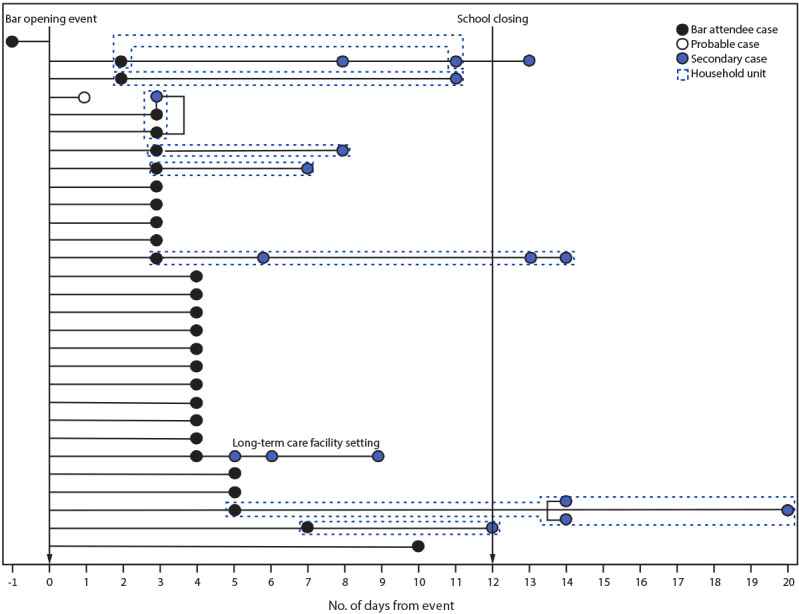
Bar attendee* (N = 29) and secondary^†^ (N = 17) confirmed^§^ and probable^¶^ COVID-19 cases associated with a local bar opening event, by timing of specimen collection relative to event — Illinois, February 2021 **Abbreviation:** NAAT = nucleic acid amplification test. * Onset of COVID-19–like symptoms or receipt of a positive SARS-CoV-2 test result within 14 days of the bar opening event in a bar patron or employee who reported attending the event and who had no previous identified epidemiologic link to a COVID-19 case outside that setting. ^†^ Positive SARS-CoV-2 NAAT or antigen test result received by a close contact of a person with an event-associated case. ^§^ Positive SARS-CoV-2 NAAT or antigen test result. ^¶^ COVID-19–like symptoms in a person with an epidemiologic link to the bar opening event but no laboratory confirmation of infection.

**TABLE:** Number and percentage of COVID-19 cases (N = 46) associated with a local bar opening event, by demographic and clinical characteristics — Illinois, February 2021

Characteristic	No. (%)				
	Bar attendee cases (N = 29)	Secondary cases			
		Total (N = 17)	Household contacts (n = 12)	Long-term care facility contacts (n = 3)	School-related contacts (n = 2)
**Classification**					
Bar employee	3 (10.3)	NA	NA	NA	NA
Bar patron	26 (89.7)	NA	NA	NA	NA
Adult household contact	NA	7 (41.2)	7 (58.3)	NA	NA
School-aged child (aged 10–16 yrs) contact	NA	7 (41.2)	5 (41.7)	NA	2 (100)
LTCF staff member contact	NA	1 (5.9)	NA	1 (33.3)	NA
LTCF resident contact	NA	2 (11.8)	NA	2 (66.7)	NA
**Case status**					
Symptomatic confirmed*	24 (82.8)	13 (76.5)	11 (91.7)	1 (33.3)	1 (50.0)
Symptomatic probable^†^	1 (3.4)	0 (—)	0 (—)	0 (—)	0 (—)
Asymptomatic confirmed^§^	4 (13.8)	4 (23.5)	1 (8.3)	2 (66.7)	1 (50.0)
Hospitalized^¶^	0 (—)	1 (5.9)	0 (—)	1 (33.3)	0 (—)
**Age group, yrs**					
<18	0 (—)	7 (41.2)	5 (41.7)	0 (—)	2 (100)
18–44	22 (75.9)	4 (23.5)	3 (25.0)	1 (33.3)	0 (—)
45–54	5 (17.2)	1 (5.9)	1 (8.3)	0 (—)	0 (—)
55–64	2 (6.9)	3 (17.6)	3 (25.0)	0 (—)	0 (—)
≥65	0 (—)	2 (11.8)	0 (—)	2 (66.7)	0 (—)
**Sex**					
Male	19 (65.5)	8 (47.1)	5 (41.7)	1 (33.3)	2 (100)
Female	10 (34.5)	9 (52.9)	7 (58.3)	2 (66.7)	0 (—)
Race/Ethnicity**					
Hispanic/Latino	0 (—)	0 (—)	0 (—)	0 (—)	0 (—)
White, non-Hispanic	23 (79.3)	16 (94.1)	12 (100)	2 (66.7)	2 (100)
Black, non-Hispanic	0 (—)	0 (—)	0 (—)	0 (—)	0 (—)
Multiracial/Other race	0 (—)	0 (—)	0 (—)	0 (—)	0 (—)
Unknown	6 (20.7)	1 (5.9)	0 (—)	1 (33.3)	0 (—)

**Abbreviations:** LTCF = long-term care facility; NA = not applicable; NAAT = nucleic acid amplification test.

* Positive NAAT or antigen test result received by a symptomatic person.

^†^ COVID-19–like symptoms in a person with an epidemiologic link to the bar opening event but no laboratory confirmation of infection.

^§^ Positive NAAT or antigen test result received by an asymptomatic person.

^¶^ Hospitalized within 14 days of the positive SARS-CoV-2 test result.

** Persons for whom ethnicity was missing or ethnicity was reported but race was missing were categorized as having unknown race/ethnicity.

**Secondary community cases.** After the bar opening event, at least 71 close contacts of persons with bar attendee COVID-19 were reported; among these, 37 (52.1%) received testing, 17 (45.9%) of whom received a positive test result within 14 days of the contact. Two persons with secondary COVID-19 cases were school-related contacts of persons with bar attendee COVID-19, three were LTCF contacts, and 12 were household contacts. Among the 17 persons with secondary cases of confirmed COVID-19, 13 were symptomatic, with symptom onset dates ranging from 3 to 11 days after the event. Median age was 28 years (range = 10–71 years), and nine persons were female.

One bar attendee with COVID-19 reported the onset of a runny nose 2 days after the event and reported 26 close contacts at school during indoor sports practice and in-person school instruction. Two student athletes who were close contacts of this person subsequently received COVID-19 diagnoses 8 and 13 days after the event. Local health department officials were notified by a school official that the school district would close for 2 weeks beginning February 18 because 13 staff members were in isolation, in quarantine, or absent because their own child was quarantined.

One bar attendee who worked at an LTCF as a certified nursing assistant was asymptomatic and received a positive test result during routine COVID-19 testing at the facility 4 days after the event. After receipt of the positive test result, all LTCF residents and staff members in the facility were tested; three secondary cases (one in a staff member and two in residents) in persons who were close contacts of the bar attendee with COVID-19 were identified in the facility 5–9 days after the event. One resident with a secondary case was hospitalized on February 20, within 14 days of the positive test result, and was discharged the same day. None of the four persons in the LTCF with bar attendee or secondary COVID-19 had received a COVID-19 vaccination; all LTCF staff members and residents had been previously offered the vaccine.

By February 26, 12 household contacts in eight different households had received positive SARS-CoV-2 test results, including five school-aged children. Local health department staff members interviewed household contacts to assess exposures. Secondary household cases were linked to nine (31.0%) of 29 bar attendee cases. Eleven persons were symptomatic, and cases were confirmed by NAAT or antigen testing. No household contacts with COVID-19 were hospitalized.

## Discussion

An event held to celebrate a bar opening led to an outbreak among bar patrons and employees and was the likely source of subsequent COVID-19 transmission among household members, LTCF residents and staff members, and school athletes, leading to 46 cases, hospitalization of an LTCF resident, and a school closure affecting 650 children. Attendees included one person with an asymptomatic infection who received a COVID-19 diagnosis the day before the event and four symptomatic persons who subsequently received a COVID-19 diagnosis after the event. Asymptomatic persons are estimated to account for approximately 40%–45% of infections (*3*); the high percentage (82.6%) of symptomatic persons who were linked to the bar opening event suggests that the total number of cases in this outbreak was higher than reported and highlights the need for increased testing and reporting through contact tracing, in combination with isolation and quarantine, to promptly reduce transmission. As community businesses begin to reopen, these findings underscore the importance of businesses and individuals adhering to public health prevention and mitigation guidelines to reduce additional community transmission, including isolation after receipt of a COVID-19 diagnosis and while experiencing COVID-19–like symptoms, even as vaccination efforts expand.

Similar gatherings that involve eating or drinking, such as on-premises dining at restaurants, weddings, and night clubs, have been associated with increased risk for acquiring COVID-19 and have the potential to become super-spreading events for SARS-CoV-2 infection (*4*–*8*). This investigation further demonstrates that inconsistent mask use and inadequate physical distancing in an indoor environment can increase transmission risk** (*9*,*10*). According to CDC’s COVID-19 guidelines for restaurants and bars, reducing patron capacity, ensuring adequate room air ventilation, prioritizing outdoor seating, and promoting behaviors such as staying at home when ill, washing hands often, wearing masks, and maintaining physical distance are important strategies to reduce the spread of SARS-CoV-2 infection (*1*).

The findings in this report are subject to at least four limitations. First, interviews were voluntary, and many community members were reluctant to disclose contacts or additional details about themselves, including their occupation. Therefore, the number of cases described in this report is likely lower than the actual number of bar attendee and secondary cases associated with the event. Second, it is unlikely that all asymptomatic cases were counted, and not all contacts were tested; therefore, some infected contacts might have been missed. Third, information on individual-level behaviors such as wearing masks and physical distancing was not collected from persons with cases. Finally, specimens were not available for whole genome sequencing; thus, the relationship among strains was not documented, and whether the increase in county-level incidence might be attributed to variants that spread more easily than the original SARS-CoV-2 strain cannot be determined.

Bars can play a role in community spread of COVID-19 because of limited mask use while eating or drinking and lack of consistent physical distancing. These findings show that SARS-CoV-2 transmission originating in a business such as a bar not only affects the patrons and employees of the bar but can also affect an entire community. As community businesses begin to reopen, considering additional prevention measures is important, such as limiting building occupancy levels and improving ventilation, especially in locations where consistent and correct mask wearing and physical distancing are difficult to enforce. Businesses can work with local health officials to promote behaviors and maintain environments that reduce the risk for SARS-CoV-2 transmission and develop strategies for reopening safely to prevent outbreaks in the community, such as modifying layouts and operating procedures (*1*).

**Summary**

**What is already known about this topic?**

Gatherings in settings where mask wearing and physical distancing do not occur are known to increase the spread of COVID-19.

**What is added by this report?**

Forty-six cases of COVID-19 were linked to an indoor bar opening event that occurred during February 2021 in a rural Illinois county. Event patrons were linked to secondary cases among household, long-term care facility, and school contacts, resulting in one hospitalization and one school closure affecting 650 students.

**What are the implications for public health practice?**

Opening up settings such as bars, where mask wearing and physical distancing are challenging, can affect the community. As community businesses reopen, prevention measures should be emphasized, including limiting building occupancy, improving ventilation, prioritizing outdoor seating, enforcing correct mask wearing and physical distancing, staying home when ill, and encouraging COVID-19 vaccination to reduce transmission on site and within the community.

## Footnotes

* https://www.cdc.gov/coronavirus/2019-ncov/community/large-events/considerations-for-events-gatherings.html; https://www.cdc.gov/coronavirus/2019-ncov/community/ventilation.html

†
https://www.cdc.gov/coronavirus/2019-ncov/symptoms-testing/symptoms.html

§ Close contact is defined as being within 6 ft of a person with laboratory-confirmed or probable SARS-CoV-2 infection for a cumulative total of ≥15 minutes in a 24-hour period. https://www.cdc.gov/coronavirus/2019-ncov/php/contact-tracing/contact-tracing-plan/appendix.html#contact

¶ 45 C.F.R. part 46.102(l)(2), 21 C.F.R. part 56; 42 U.S.C. Sect. 241(d); 5 U.S.C. Sect. 552a; 44 U.S.C. Sect. 3501 et seq.

**
https://www.medrxiv.org/content/10.1101/2020.04.04.20053058v1
